# Clinical and inflammatory biomarkers of inflammatory bowel diseases are linked to plasma trace elements and toxic metals; new insights into an old concept

**DOI:** 10.3389/fnut.2022.997356

**Published:** 2022-12-08

**Authors:** Charalampia Amerikanou, Sotirios Karavoltsos, Aristea Gioxari, Dimitra Tagkouli, Aikaterini Sakellari, Efstathia Papada, Nick Kalogeropoulos, Alastair Forbes, Andriana C. Kaliora

**Affiliations:** ^1^Department of Nutrition and Dietetics, School of Health Science and Education, Harokopio University, Athens, Greece; ^2^Laboratory of Environmental Chemistry, Department of Chemistry, National and Kapodistrian University of Athens, Athens, Greece; ^3^Department of Nutritional Science and Dietetics, University of Peloponnese, Tripolis, Greece; ^4^Division of Medicine, University College London, London, United Kingdom; ^5^Institute of Clinical Medicine, University of Tartu, Tartu, Estonia

**Keywords:** inflammatory bowel diseases, Crohn's disease, ulcerative colitis, metals, trace elements, inflammation

## Abstract

**Background:**

Inflammatory bowel diseases (IBD) are chronic immune-mediated diseases, mainly represented by Crohn's disease (CD) and ulcerative colitis (UC). Several environmental factors have been proposed to contribute to disease pathogenesis, amongst which are metals. These can affect the immune system and may be associated with IBD. The aim of the present cross-sectional study was to investigate blood levels of metals in IBD patients and to examine possible associations with clinical and inflammatory disease markers.

**Methods:**

In total, 76 CD patients, 39 UC patients and 38 healthy controls were included. Blood and stool samples were collected. Metals were quantified in plasma samples using inductively coupled plasma mass spectrometry.

**Results:**

There were more abnormalities of circulating metals in CD than in UC when compared to healthy controls. CD: Concentrations of the essential trace elements zinc and selenium were lower in CD patients than the controls. Chromium was negatively associated with serum IL-6 (Beta: −3.558, *p* = 0.011), and caesium with fecal calprotectin (Beta: −0.481, *p* = 0.038) and serum IL-10 (Beta: −1.912, *p* = 0.050). In contrast, copper was positively associated with C-reactive protein (Beta: 2.548 × 10^2^, *p* = 0.033). UC: In UC, a negative association of iron with serum myeloperoxidase levels (Beta: −1.270 × 10^3^, *p* = 0.044) was detected. Thallium, a hazardous metal, however, was positively associated with disease activity (Beta: 3.899, *p* = < 0.01).

**Conclusion:**

In conclusion, our study offers new insights into the relations of metals with IBD. Further research should focus on the evaluation of the above associations and potential underlying mechanisms.

## Introduction

The inflammatory bowel diseases (IBDs) are chronic immune-mediated diseases characterized by chronic relapsing and remitting inflammation of the gastrointestinal tract. IBD is mainly represented by Crohn's disease (CD) and ulcerative colitis (UC) (1, 2). CD affects areas of the entire gastrointestinal tract with the inflammation being segmental, asymmetrical, and transmural, whereas UC is localized to the rectum and to more proximal segments of the colon, in a continuous way (3, 4).

Although the pathophysiology of IBD has not yet been clearly elucidated, it has been hypothesized that there is an interplay between genetic background, multiple environmental factors, dysbiosis of the gut microbiota, and immune responses. C*hronological and geographical changes* in the epidemiology of IBD support the critical role of the environment in the pathogenesis (5). Environmental factors may include diet, smoking, a lack of breastfeeding, urbanization, and use of antibiotics. Environmental pollution can also serve as a risk factor for IBD, and exposure to SO_2_ and NO_2_ have been shown to increase the risk for UC and CD, respectively (6).

The heavy metals—which occur naturally in earth's crust–are metallic elements with relatively high density and have the potential to be toxic or poisonous even at low concentrations due to bioaccumulation. Examples include mercury, cadmium, arsenic, chromium, thallium, and lead. These enter the human body in small amounts through the alimentary or respiratory tracts, and if retained cannot be degraded or destroyed. Other metals such as copper, selenium, and zinc are essential to human metabolism as trace elements, although at high concentrations these too can prove to be toxic.

Heavy metal poisoning may be caused by consumption of contaminated drinking-water, by inhaling air near sources of emission, or by ingestion of contaminated food. Urbanization and industrialization have increased solid wastes generation with consequent loading of soil and groundwater with heavy metals; this can have a serious adverse impact on human health (7, 8). Several metals affect the immune system and have been associated with inflammatory diseases, including IBD (9–12). Mercury interferes with the intestinal epithelium, inhibits the production of enzymes, disrupts the gut microbiota and contributes to IBD pathogenesis (13). Long-term exposure to cadmium, which is retained in the gastrointestinal tract, can exacerbate colitis by disrupting the intestinal barrier (14). However, cadmium, and to some extent lead, paradoxically have been shown to reduce the severity of symptoms caused by TNBS and DSS induced colitis in a significant and dose-dependent manner (15), with arsenic even been used therapeutically in colitis (16).

The trace elements essential to man also exert major effects on the function of the immune system. For example, zinc and copper are components of superoxide dismutase (SOD) and thereby contribute to the decrease of oxidative stress (17). IBD patients with zinc deficiency are more likely to have adverse disease-specific outcomes such as hospitalization, surgery, and disease-related complications (18). In mice, manganese deficiency disrupts intestinal barrier function and increases susceptibility to colitis due to aberrant exposure of the host immune system to the gut microbiome (19). As a member of the NF-kB inhibitor family, chromium has been shown to reduce CRP, TNF-a, and interleukin (IL)-6 levels in multiple studies (20).

The role of metals in IBD is well established; systemic inflammatory response is responsible for deficiencies in trace elements and subsequent clinical impact, or chronic exposure to heavy metals is associated with IBD pathogenesis (21). Nevertheless, several metals are known to regulate or aggravate inflammatory responses, either by inhibiting the production of proinflammatory cytokines or by contributing to the initiation of inflammatory processes. However, not many studies have evaluated the associations between circulating metals and disease markers that may provide new insights into the role of metals in IBD pathogenesis. So, the primary hypothesis of this study was that circulating levels of metals in IBD patients are differentially expressed compared to those of healthy individuals and this altered expression is reflected in the associations with inflammatory disease markers. The differences might be due either to malabsorption attributed to the clinical manifestation of the disease or to higher exposure to hazardous metals. Thus, the aim of this study was to explore the circulating metals in IBD patients as compared to healthy individuals, and to examine their interplay with a wide range of clinical and inflammatory disease markers.

## Materials and methods

### Ethics

All enrolled patients and healthy participants signed an informed consent after being provided with a detailed information leaflet describing the study procedures. The study followed the principles of Declaration of Helsinki and was approved by the Ethics Committee of Harokopio University (49/29-10-2015).

### Participants

The present cross-sectional study included 76 patients with CD, 39 patients with UC and 38 healthy controls (HC; both males and females), and took place in Athens, Greece. Enrolment was stimulated through announcement to the Hellenic Society of CD and UC patients and patients were from different regions throughout Greece. The IBD patients had a confirmed endoscopic diagnosis and were enrolled in the IBD-GR cohort (22, 23). Exclusion criteria were: age < 18 years; positive stool culture for enteric pathogens; bowel surgery ≤ 3 months prior to screening; short bowel syndrome; intra-abdominal abscess or fistula with clinical or radiological evidence of an associated abscess; ileostomy or colostomy; enteral or parenteral nutrition; alcohol or drug abuse; vitamin or inorganic supplement use ≤ 6 months prior to screening; a vegan or macrobiotic diet ≤ 5 years prior to screening; any malignancy ≤ 1 year- or cancer survivors ≤ 10 years prior to screening; serious cardiovascular disease, peptic ulcer, pregnancy or lactation.

### Medical, anthropometric and dietary assessment

Detailed medical history and disease characteristics were obtained by one experienced gastroenterologist. Patients were classified clinically as being in relapse or in remission. The Partial Mayo Score (PMS) and the Harvey–Bradshaw Index (HBI) were used to quantify the disease activity in UC and CD, respectively (24, 25), and the Inflammatory Bowel Disease Questionnaire (IBDQ) was administered to assess quality of life (26). Body weight was measured to the nearest 0.1kg, height was measured with a standard stadiometer to the nearest millimeter and body mass index (BMI) was calculated. Dietary intake was assessed by an experienced dietitian using the 24-h recall record and the software package Nutritionist Pro™ (Axxya Systems, Stafford, TX, USA).

### Biological sample collection

Twenty ml of blood were collected after overnight fasting and were centrifuged at 3,000 rpm for 10 min at 4°C for plasma and serum isolationSerum was used for routine biochemical analysis and quantification of biomarkers of inflammation and oxidative stress. Plasma was used for evaluation of the metals. Stool samples were collected using a stool preparation system filled with extraction buffer IDK Extract^®^ (Immundiagnostik, AG) for measurement of fecal inflammatory biomarkers.

### Biochemical analyses

Serum iron (Fe), albumin, bilirubin, glucose, total cholesterol, high-density lipoprotein (HDL), low-density lipoprotein (LDL), triglycerides, urea and plasma fibrinogen, amylase, lactate dehydrogenase (LDH), glutamic–oxaloacetic transaminase (SGOT), glutamic–pyruvic transaminase (SGPT), γ-glutamyl transferase (γ-GT), alkaline phosphatase (ALP) and C-reactive protein (CRP) were quantified with an automatic biochemical analyser (Cobas 8000 analyser, Roche Diagnostics GmbH, Mannheim, Germany). Vitamin D was measured with an automated immunoassay system Cobas e801 (Roche Diagnostics, Mannheim, Germany).

### Inflammatory and oxidative stress biomarkers assessment

Interleukin-6 (R&D Systems, Inc., Minneapolis, USA), IL-10 (OriGene Technologies, Inc., Maryland, USA), IL-17A (Boster Biological Technology, Pleasanton, CA, USA), oxidized low-density lipoprotein (ox-LDL; Mercodia, AB, Uppsala, Sweden), myeloperoxidase (MPO; Thermo Fisher Scientific Inc, Massachusetts, USA) were measured in serum applying sandwich ELISA. Calprotectin, lysozyme, defensin and lactoferrin were quantified in stool samples applying sandwich ELISA (Immundiagnostik, AG, Bensheim, Germany). All measurements were performed in duplicate in all patients.

### Inductively coupled plasma mass spectrometry

All plastic materials that came into contact with the blood samples were previously washed thoroughly, soaked in dilute HNO_3_ (Merck, Darmstadt, Germany), and rinsed with ultrapure water of 18.2 MΩ cm (Millipore, Bedford, MA, USA). Micropipettes used for the dilution of the samples were calibrated regularly. For the preparation of all solutions required, class A volumetric glassware was used. Samples were digested using a mixture of HNO_3_ (suprapur 65%; Merck) and H_2_O_2_ (suprapur 30%; Merck), according to the procedures described by Batáriová et al. and Jin et al., slightly modified (27, 28). The digested samples were analyzed by inductively coupled plasma mass spectrometry (ICP-MS; Thermo Scientific ICAP Qc, Waltham, MA USA). Measurements were carried out in a single collision cell mode, with kinetic energy discrimination (KED) using pure He. Matrix-induced signal suppressions and instrumental drift were corrected by the use of internal standards (^45^Sc, ^103^Rh). Plasma samples were analyzed in duplicate and the average of the two measurements was used for statistical analysis. The limits of detection (LOD), calculated according to US EPA (29), varied from 0.03 μg/L for cesium and thallium to 0.8 for iron. For statistical calculations, values below the minimum were assigned the method detection limit divided by √2.

### Statistical analysis

Data are presented as mean ± standard deviation (SD) or median and interquartile range (IQR). Normal distribution was assessed with Kolmogorov–Smirnov test. Chi-square test was used for the comparison of proportions. Student's *t*-test and Mann–Whitney *U*-test were used for the comparisons of means between two independent groups. For the comparisons of means for more than two groups we used ANOVA or Kruskal–Wallis *H*-test, applying *posthoc* comparison using Bonferroni's correction. Spearman's correlation test was used for the correlation analysis, whereas linear regression models were applied for the exploitation of the relationships of parameters showing a significant bivariate correlation. Parameters that were not normally distributed were log transformed where needed. Statistical analysis was conducted with the SPSS software (SPSS for Windows, version 21.0, SPSS Inc., Chicago, IL, USA).

## Results

Demographic and clinical characteristics of the study population are presented in Table 1. CD, UC patients and HC were well matched for age, sex and BMI. Disease duration and IBDQ scores did not differ between CD and UC patients. HBI and PMS differed significantly according to clinically assessed disease activity (HBI: 7.3 ± 2.4 vs. 2.3 ± 1.4 with *p* < 0.01, PMS: 4.1 ± 1.9 vs. 0.7 ± 0.5 with *p* < 0.01 in active vs. inactive CD and UC patients, respectively), and IBDQ was highly negatively correlated with both disease activity indices (rho: −0.559, *p* < 0.01 and rho: −0.639, *p* < 0.01 for HBI and PMS, respectively). Levels of biochemical, inflammatory and oxidative stress biomarkers in the two patient groups are shown in Supplementary Tables S1, S2.

**Table 1:** Demographic and clinical characteristics of the participants.

	**CD**	**CD relapse**	**CD remission**	**UC**	**UC relapse**	**UC remission**	**HC**	***p* ^1^**	***p* ^2^**
	**(*N* = 76)**	**(*N* = 38)**	**(*N* = 38)**	**(*N* = 39)**	**(*N* = 18)**	**(*N* = 21)**	**(*N* = 38)**		
Sex (M/F)	42/34	18/20	24/14	17/22	7/11	10/11	16/22	0.151	0.335
Age (years) (mean ± SD)	38.7 ± 14.0	40.6 ± 16.7	36.8 ± 10.6	39.3 ± 11.8	41.3 ± 14.4	37.6 ± 9.0	33.0 ± 12.2	0.062	0.096
BMI (kg/m^2^) (mean ± SD)	23.5 ± 4.4	23.5 ± 4.8	24.3 ± 3.8	24.6 ± 5.4	24.6 ± 6.2	24.6 ± 4.8	24.9 ± 4.5	0.245	0.312
Smoking (Yes/No)	28/48	14/24	14/24	13/26	4/14	9/12	N/A	0.367	0.403
Disease duration (years) (mean ± SD)	11.3 ± 8.1	11.9 ± 9.1	10.7 ± 7.1	9.4 ± 6.9	9.4 ± 7.2	9.4 ± 6.9	–	0.214	0.575
Disease location (*N*)									
Colonic	2	0	2	3	1	2	–		
Ileo-colonic	30	14	16	0	0	0	–		
Ileal	24	15	9	0	0	0	–		
Left-sided	3	2	1	10	5	5	–		
Pancolitis	1	0	1	20	10	10	–		
Other	16	7	9	6	3	3	–		
IBDQ score (mean ± SD)	162.6 ± 27.8	148.0 ± 22.4	176.4 ± 25.5	162.9 ± 35.0	145.6 ± 31.3	177.7 ± 31.5	–	0.966	**< 0.01**
HBI (mean ± SD)	4.8 ± 3.2	7.3 ± 2.4	2.3 ± 1.4	–	–	–	–	**< 0.01**	
PMS (mean ± SD)	–	–	–	2.3 ± 2.1	4.1 ± 1.8	0.7 ± 0.5	–	**< 0.01**	

CD, Crohn's disease; UC, ulcerative colitis; HC, healthy control; BMI, body mass index; IBDQ, Inflammatory Bowel Disease Questionnaire; HBI, Harvey Bradshaw index; PMS, Partial Mayo score.

Student's t-test was used to analyse differences between two independent groups and One-way ANOVA between more than two independent groups. Posthoc comparison was conducted using Bonferroni's correction. Chi-squared test was used for the comparison of proportions. Difference was considered significant at p-value < 0.05. p^1^, comparison between CD, UC and HC; p^2^, comparison between CD relapse, CD remission, UC relapse, UC remission, and HC (when applicable). Values in bold indicate significant differences.

Table 2 and Supplementary Table S3 present the different levels of metals in the three examined study groups (CD, UC, and HC) and among different disease activity categories and HC, respectively. UC patients exhibited lower levels of manganese, nickel, zinc, selenium and strontium compared with HC. CD patients had significantly lower levels only of selenium compared to HC, and higher levels of strontium than patients with UC. Nickel was elevated in CD patients in relapse, compared to UC patients in remission, and lower in UC remission compared with HC.

**Table 2:** Levels of trace metals (μg/L) in the plasma of CD, UC patients and HC.

	**CD**	**UC**	**HC**	***p* ^1^**	***p* ^2^**
	**(*N* = 76)**	**(*N* = 39)**	**(*N* = 38)**		
Vanadium	0.23 ± 0.92	0.33 ± 0.82	0.08 ± 0.54	0.928	ns
Chromium	2.6 ± 3.8	1.6 ± 5.1	2.3 ± 3.4	0.148	ns
Manganese	1.8 ± 4.3	1.4 ± 3.0^a^	2.4 ± 2.4^a^	**0.042**	**a** **=** **0.041**
Iron	1451 ± 794	1258 ± 935	1341 ± 756	0.269	ns
Cobalt	0.49 ± 0.74	0.26 ± 0.69	0.34 ± 0.72	0.549	ns
Nickel	2.9 ± 10.4	1.2 ± 12.3^a^	5.5 ± 7.4^a^	**0.017**	**a** **=** **0.014**
Copper	1021 ± 389	951 ± 272	909 ± 226.7	0.851	ns
Zinc	818 ± 1412	570 ± 846^a^	968 ± 871^a^	**0.027**	**a** **=** **0.022**
Arsenic	0.89 ± 1.4	0.44 ± 1.4	0.71 ± 1.2	0.458	ns
Selenium	50 ± 39^a^	44 ± 41^b^	77 ± 30^a,b^	**0.009**	**a** **=** **0.018**, **b** **=** **0.020**
Rubidium	542 ± 227	474 ± 120	488 ± 95	0.469	ns
Strontium	37 ± 19^a^	29 ± 12^a,b^	35 ± 20^b^	**0.024**	**a** **=** **0.041**, **b** **=** **0.053**
Cadmium	0.32 ± 0.69	0.33 ± 0.75	0.19 ± 0.36	0.096	ns
Cesium	0.68 ± 0.54	0.65 ± 0.27	0.64 ± 0.43	0.675	ns
Thallium	0.02 ± 0.02	0.02 ± 0.00	0.02 ± 0.05	0.578	ns

Values are presented as mean ± standard deviation of the mean. Data are presented as median (interquartile range).

p^1^, differences between groups were analyzed using Kruskal–Wallis test. p^2^, adjusted p-values after Bonferroni correction for pairwise comparisons. Difference was considered significant at p-value < 0.05. Values sharing the same superscript differ significantly.

ns, non-significant. Values in bold indicate significant differences.

The results of the correlation analysis between metals levels and parameters that reflect disease activity in CD and UC patients are presented in Supplementary Tables S4, S5, respectively. A linear regression analysis was applied to address the significant associations presented above (Table 3). We applied three approaches, one unadjusted, one adjusted for age, sex and BMI (Model 1), and one adjusted for age, sex, BMI, smoking, disease activity, location, duration, nutritional supplementation, and dietary intake of the respective metal if available from Nutritionist Pro analysis (Model 2). The relationships with the statistically significant results in all three modes are presented in Table 3. In CD, chromium was negatively associated with log IL-6 (Beta: −3.558, *p* = 0.011), and cesium with log calprotectin (Beta: −0.481, *p* = 0.038) and log IL-10 (Beta: −1.912, *p* = 0.050). Copper was positively associated with log CRP (Beta: 2.548 × 10^2^, *p* = 0.033). In UC, iron was negatively associated with log myeloperoxidase (MPO; Beta: −1.270 × 10^3^, *p* = 0.044) and thallium positively with log PMS (Beta: 3.899, *p* = < 0.01).

**Table 3:** Regression analysis addressing the associations between trace metals and other data where there were significant correlations (only relationships with significant results in Model 2 are presented).

	**Unadjusted**		**Model 1^a^**		**Model 2^b^**	
	**Beta**	***P*-value**	**Beta**	***P*-value**	**Beta**	***P*-value**
**CD**						
**Chromium**						
log IL-6	−2.033	**0.004**	−1.862	**0.013**	−3.558	**0.011**
**Copper**						
log CRP	1.696 × 10^2^	**0.004**	2.020 × 10^2^	**< 0.01**	2.548 × 10^2^	**0.033**
**Cesium**						
log calprotectin	−0.328	**0.006**	−0.389	**0.003**	−0.481	**0.038**
log IL-10	−1.514	**0.008**	−1.945	**0.002**	−1.912	**0.050**
**UC**						
**Iron**						
logMPO	−8.645 × 10^2^	0.033	−9.226 × 10^2^	0.025	−1.270 × 10^3^	**0.044**
**Thallium**						
logPMS	0.444	**0.032**	0.500	**0.023**	3.899	**< 0.01**

Fe, iron; CRP, C-Reactive Protein; IL, Interleukin; MPO, myeloperoxidase; PMS, Partial Mayo score.

Significance level was set at *p* < 0.05.

a Model 1: adjusted for age, sex, BMI.

b Model 2: adjusted for age, sex, BMI, smoking, disease activity, location, duration, nutritional supplementation, and dietary intake of the respective metal if available from Nutritionist Pro analysis.

CRP, IL-6, IL-10, calprotectin, MPO, and PMS were not normally distributed therefore their logarithm was used for the regression. Values in bold indicate significant associations.

## Discussion

The IBDs are complex conditions where genetics, immune response, gut microbiota and environmental factors overlap to manifest these pathologies. Since approximately two-thirds of IBD patients show no identifiable genetic defect, it is suggested that other factors, such as the environment may play a critical role (30). Heavy metals are naturally occurring in the environment and some of them are essential as trace elements in the human body. However, their alarmingly increased concentrations firstly in the habitat and secondly in the living organisms entails hazards that are sustained due to the nature of these metals and their accumulation in the ecosystems.

The role of heavy metals in the establishment and progression of IBD and in the maintenance of gut health has been explored. However, only a few studies have addressed the potential relationship of their circulating levels in IBD patients with parameters reflecting disease activity, such as that of Stojsavljević et al. (31) where they explored the circulating levels of essential and toxic trace elements in patients with CD and potential correlations of metal ratios with CRP and calprotectin. To the best of our knowledge, this is the first study that examines the associations of metals with disease activity indices (such as PMS, HBI, inflammatory and oxidative stress biomarkers), more importantly including regression analysis and adjustment for strong potential confounders. Also this is the first study exploring the above relations in a Greek population.

Manganese, nickel, zinc, selenium, and strontium were generally lower in UC patients than in HC and selenium was lower in CD patients. Strontium was higher in CD than in UC. Nickel was elevated in patients with active CD compared to those in remission, but was lower in inactive CD. Our results are therefore broadly in line with previous research evidence where this exists. Particularly in the case of zinc, the circulating element is largely bound to albumin and so when albumin concentrations decrease, total zinc concentrations would also be expected to decrease. Other trace elements have specific high affinity carrier proteins such as transferrin (iron) and caeruloplasmin (copper) which may themselves be acute phase reactants. In addition, several trace elements and carrier proteins have been found to cause negative inflammatory responses, and a low level of these analytes in inflammatory diseases is not necessarily indicative of nutritional deficiency (32). In the case of CD, there are several processes, other than reduced dietary intake, which can result in apparent trace element deficiency, including impaired mucosal absorption, increased excretion, and hypoalbuminaemia (33). Mucosal malabsorption and nutritional deficiencies of trace elements are nonetheless likely to play a central role.

Zinc is absorbed in the small intestine and plays an important role as an enzyme cofactor involved in immune function (18). Its deficiency is common in IBD patients.

Manganese is essential for cell function, as it serves as a cofactor in reactions catalyzed by several enzymes, some of them implicated in defense against oxidative stress and therefore possibly in IBD pathogenesis as well. Experimental mice with manganese deficiency exhibited impaired intestinal tight junctions and disturbed intestinal permeability, while they showed increased tolerance to colitis when supplemented with manganese (34). In line with our results selenium and manganese were found at significantly lower levels in the hair of pediatric patients with CD and UC than in healthy controls (35). The inverse association of selenium with IBD is better established, with several selenoproteins mediating gut inflammation and activating immune response (36).

Nickel levels have not previously been evaluated in IBD, but several studies refer to the disrupting role of nickel in gut microbiota eubiosis (37). Our results agree in part with these as nickel levels were higher in active CD compared to inactive UC (although controls had higher levels than UC patients).

Strontium is known for its anti-inflammatory role and is used for the treatment of inflammatory conditions as it inhibits TNF-α induced NFκB signal transduction. It has also been proposed as a treatment option for UC, since strontium chloride suppressed inflammation in a rat ulcerative colitis model (38).

Regression analysis revealed interesting relationships between plasma metals and disease-related factors in our cohort. In CD, chromium was negatively associated with serum IL-6, and cesium with fecal calprotectin and serum IL-10. Copper was positively associated with CRP.

Chromium has a well-known anti-inflammatory role, as it inhibits NFκB activation and its supplementation has been linked to reductions of serum CRP, TNF-α, and IL-6 in a meta-analysis of randomized controlled trials (20, 39). Also, chromium supplementation improved histological findings, upregulated IL-10, and downregulated TNF-α, and IFN-γ in experimental colitis (40).

Although there was no prior evidence for a role of cesium in IBD, this element is usually found at lower levels in other inflammatory disorders when compared with healthy controls and has been shown to exhibit anti-inflammatory and antioxidant effects (41–43). The apparently contradictory findings in our study, cesium correlating with lower levels of calprotectin and IL-10, are in line with previous research findings that suggested an immunomodulatory role of cesium regulating both pro- and anti-inflammatory cytokines (43).

The role of copper in IBD is well-documented as it affects the immune response, by serving as an enzyme cofactor in several pathways, such as antioxidant mechanisms. Inflamed mucosa in IBD patients seems to bear lower levels of the copper-containing protein superoxide dismutase, known for its antioxidant activity, suggesting a decreased protection against oxidation (44). Similarly, in our population, copper was positively associatedwith CRP.

Regression analysis in UC revealed a negative association of iron with MPO. Iron deficiency is very common in IBD patients due to inadequate dietary intake, malabsorption and chronic blood loss and has been associated with disease activity, while increased CRP levels have been linked with increased prevalence of anemia in patients with IBD (45). In the present study, iron levels in blood were within the normal range in both UC and CD patients, but in UC iron was associated with lower MPO, an enzyme with bactericidal function known for its upregulation in IBD and correlation with disease activity markers, supporting a crucial role of iron in the disease (46).

Finally, thallium was positively associated with the PMS in UC. Thallium is one of the main hazardous metals according to the World Health Organization and can affect several organs, including the gastrointestinal tract. Its serum levels correlate with TNF-α, and IL-6 in pregnant women, whereas its urine levels correlate with CRP in smokers (47, 48). In our study thallium correlated with PMS, a disease activity index that correlates well with several disease markers.

The relative small sample size, as well as the lack of recent endoscopy to determine the disease activity are the main limitations of our study, but they are compensated by several strengths. All the tools that have been used (disease activity indices, dietary analysis) are validated and the methodologies for biomarker assessment offer high sensitivity and specificity. ICP-MS is a well-established high-throughput technique with very low detection limits and in our study was able to detect 16 metals simultaneously. Of course, as the above data have been collected in a cross-sectional design and a causal relationship between heavy metals and IBD cannot be speculated, the results should be interpreted with caution. To the best of our knowledge this is the first study that quantifies such a wide variety of metals in the plasma of IBD patients.

## Conclusions

The contribution of circulating metals to IBD pathogenic mechanisms is interesting and is gaining increased attention. There have not been adequate data on the levels of metals in IBD patients, nor on the association with disease activity and inflammation, and our study offers new insights into these relationships. Further research should focus on further evaluation of the associations demonstrated and their underlying mechanisms.

## Data availability statement

The original contributions presented in the study are included in the article/Supplementary material, further inquiries can be directed to the corresponding authors.

## Ethics statement

The studies involving human participants were reviewed and approved by Ethics Committee of Harokopio University (49/29-10-2015). The patients/participants provided their written informed consent to participate in this study.

## Author contributions

AK conceptualized and designed the study. CA, AG, EP, SK, DT, and AS were involved in the investigation. CA performed the analysis and drafted the initial version of the manuscript. AG, SK, DT, AS, EP, NK, AF, and AK reviewed the manuscript. All authors approved the final version.
