# Supplementary material for: Clinical and inflammatory biomarkers of inflammatory bowel diseases are linked to plasma trace elements and toxic metals; new insights into an old concept
Source: Front Nutr. 2022 Dec 8;9:997356. doi: 10.3389/fnut.2022.997356 (PMC9780073; doi:10.3389/fnut.2022.997356)
Supplement: Supplementary file 1 [file Table_1.docx]

**Supplementary Table S1**. Levels of biochemical parameters between the entities of CD and UC

|  | **CD (N=76)** | **UC (N=39)** | **P** |
| --- | --- | --- | --- |
| **Amylase (iu/L)** | 67.0 (24.0) | 62.5 (33.0) | 0.954 |
| **Fibrinogen (mg/dL)** | 273.0 (105.0) | 240.5 (68.0) | 0.056 |
| **Iron (μg/dL)** | 58.0 (45.0) | 56.5 (29.3) | 0.682 |
| **Bilirubin (mg/dL)** | 0.3 (0.2) | 0.3 (0.2) | 0.943 |
| **Glucose (mg/dL)** | 89.0 (17.0) | 83.0 (24.5) | 0.990 |
| **Urea (mg/dL)** | 30.0 (10.0) | 32.5 (12.8) | 0.067 |
| **Total cholesterol (mg/dL)** | 169.0 (55.0) | 188.0 (58.3) | **0.007** |
| **HDL (mg/dL)** | 49.0 (21.0) | 54.0 (26.8) | 0.290 |
| **LDL (mg/dL)** | 87.0 (48.0) | 105.5 (39.0) | **0.006** |
| **Triglycerides (mg/dL)** | 95.0 (70.0) | 93.5 (57.8) | 0.609 |
| **Albumin (g/dL)** | 4.3 (0.7) | 4.4 (0.3) | 0.189 |
| **SGOT (iu/L)** | 15.0 (8.0) | 16.0 (4.8) | 0.595 |
| **SGPT (iu/L)** | 15.0 (13.0) | 14.0 (10.8) | 0.687 |
| **GGT (iu/L)** | 16.0 (12.0) | 14.0 (9.8) | 0.333 |
| **Alkaline phosphatase (iu/L)** | 61.0 (26.0) | 63.5 (26.3) | 0.953 |
| **Lactate dehydrogenase (U/L)** | 146.0 (41.0) | 150.0 (35.5) | 0.201 |
| **Vitamin D3 (pg/mL)** | 20.4 (10.3) | 21.4 (11.3) | 0.911 |

HDL: high-density lipoprotein, LDL: low-density lipoprotein, SGOT: serum glutamic-oxaloacetic transaminase, SGPT: serum glutamic-pyruvic transaminase, GGT: gamma-glutamyl transpeptidase

Data are presented as median (interquartile range). Differences between CD and UC patients were analysed using Mann–Whitney U test. Difference was considered significant at p < 0.05.
