# Supplementary material for: Clinical and inflammatory biomarkers of inflammatory bowel diseases are linked to plasma trace elements and toxic metals; new insights into an old concept
Source: Front Nutr. 2022 Dec 8;9:997356. doi: 10.3389/fnut.2022.997356 (PMC9780073; doi:10.3389/fnut.2022.997356)
Supplement: Supplementary file 2 [file Table_2.docx]

**Supplementary Table S2**. Levels of inflammatory and oxidative stress biomarkers between the entities of CD and UC

|  | **CD (N=76)** | **UC (N=39)** | **P** |
| --- | --- | --- | --- |
| **CRP (mg/L)** | 2.6 (11.3) | 1.8 (3.4) | 0.057 |
| **IL-6 (pg/mL)** | 9.9 (23.8) | 3.0 (5.4) | 0.072 |
| **IL-10 (pg/mL)** | 5.4 (2.2) | 10.5 (18.3) | **0.022** |
| **IL-17A (pg/mL)** | 18.3 (20.2) | 26.2 (29.5) | 0.494 |
| **Calprotectin (μg/g)** | 744.6 (1612.9) | 1276.9 (1834.8) | 0.477 |
| **Defensin (ng/g)** | 8.1 (23.2) | 7.7 (35.6) | 0.960 |
| **Lysozyme (μg/g)** | 7.6 (13.6) | 9.6 (5.8) | 0.697 |
| **Lactoferrin (μg/g)** | 60.2 (252.9) | 62.2 (197.2) | **0.027** |
| **oxLDL (U/L)** | 134.4 (66.4) | 138.0 (71.5) | 0.720 |
| **MPO (ng/mL)** | 107.4 (139.5) | 70.3 (90.5) | 0.214 |

CRP: c-reactive protein, IL-: interleukin, oxLDL: oxidized low-density lipoprotein, MPO: myeloperoxidase

Data are presented as median (interquartile range). Differences between CD and UC patients were analysed using Mann–Whitney U test. Difference was considered significant at p < 0.05.
