# Supplementary material for: Clinical and inflammatory biomarkers of inflammatory bowel diseases are linked to plasma trace elements and toxic metals; new insights into an old concept
Source: Front Nutr. 2022 Dec 8;9:997356. doi: 10.3389/fnut.2022.997356 (PMC9780073; doi:10.3389/fnut.2022.997356)
Supplement: Supplementary file 3 [file Table_3.docx]

**Supplementary Table S3**. Levels of metals (μg/L) among different disease activity categories and HC

|  | **CD relapse**  **(N=38)** | **CD remission (N=38)** | **UC relapse**  **(N=18)** | **UC remission**  **(N=21)** | **HC**  **(N=38)** | **p^1^** | **p^2^** |
| --- | --- | --- | --- | --- | --- | --- | --- |
| **Vanadium** | 0.33 (1.2) | 0.23 (0.68) | 0.43 (1.1) | 0.28 (0.78) | 0.08 (0.54) | 0.906 | ns |
| **Chromium** | 2.1 (3.8) | 3.3 (3.6) | 1.2 (2.4) | 2.0 (6.3) | 2.3 (3.4) | 0.356 | ns |
| **Manganese** | 1.2 (3.7) | 3.1 (4.0) | 0.60 (3.0) | 1.5 (3.1) | 2.4 (2.4)^a^ | 0.157 | ns |
| **Iron** | 1456 (809) | 1422 (824) | 1225 (1110) | 1264 (795) | 1341 (756) | 0.425 | ns |
| **Cobalt** | 0.39 (0.77) | 0.58 (0.75) | 0.26 (0.57) | 0.28 (0.72) | 0.34 (0.72) | 0.800 | ns |
| **Nickel** | 4.2 (16)^a^ | 2.8 (8.2) | 5.94 (12) | 1.0 (4.9)^a,^ | 5.5 (7.4)^b^ | **0.010** | **a=0.049, b=0.021** |
| **Copper** | 1067 (282) | 906 (423) | 937 (238) | 959 (304) | 909 (227) | 0.327 | ns |
| **Zinc** | 939 (1800) | 682 (830) | 598 (1573) | 420 (805) | 968 (871) | **0.028** | ns |
| **Arsenic** | 1.4 (2.6) | 0.71 (0.65) | 0.71 (1.4) | 0.33 (1.24) | 0.71 (1.2) | 0.183 | ns |
| **Selenium** | 50 (3.7)^a^ | 54 (68)^b^ | 43 (45) | 47 (40) | 77 (30)^a, b^ | **0.047** | ns |
| **Rubidium** | 473 (227) | 578 (203) | 506 (88) | 447 (96) | 488 (95) | 0.089 | ns |
| **Stronitum** | 37 (20) | 37 (19) | 32 (9.6) | 27 (14) | 35 (20) | 0.065 | ns |
| **Cadmium** | 0.29 (0.77) | 0.42 (0.69) | 0.23 (0.57) | 0.35 (0.89) | 0.19 (0.36) | 0.227 | ns |
| **Cesium** | 0.69 (0.77) | 0.68 (0.40) | 0.74 (0.31) | 0.63 (0.18) | 0.64 (0.43) | 0.770 | ns |
| **Thallium** | 0.02 (0.12) | 0.02 (0.01) | 0.02 (0.03) | 0.02 (0.00) | 0.02 (0.05) | 0.071 | ns |

Data are presented as median (interquartile range). p^1^: Differences between groups were analysed using Kruskal-Wallis test. p^2^: adjusted p values after bonferroni correction for pairwise comparisons. Difference was considered significant at p < 0.05. Values sharing the same superscript differ significantly. ns: non significant.
