# Supplementary material for: Clinical and inflammatory biomarkers of inflammatory bowel diseases are linked to plasma trace elements and toxic metals; new insights into an old concept
Source: Front Nutr. 2022 Dec 8;9:997356. doi: 10.3389/fnut.2022.997356 (PMC9780073; doi:10.3389/fnut.2022.997356)
Supplement: Supplementary file 4 [file Table_4.docx]

**Supplementary Table S4.** Significant correlations among trace metals and parameters reflecting disease activity in CD patients.

| **CD** | | **Chromium** | **Iron** | **Cobaltium** | **Nickel** | **Copper** | **Zinc** | **Arsenic** | **Selenium** | **Rubidium** | **Cesium** |
| --- | --- | --- | --- | --- | --- | --- | --- | --- | --- | --- | --- |
| IBDQ | **rho** |  |  | 0.297 |  |  |  |  | 0.278 | 0.434 |  |
|  | ***P*** |  |  | 0.012 |  |  |  |  | 0.018 | <0.01 |  |
| HBI | **rho** |  |  |  |  | 0.227 |  |  |  |  |  |
|  | ***P*** |  |  |  |  | 0.049 |  |  |  |  |  |
| CRP | **rho** | -0.327 | -0.303 |  |  | 0.431 |  |  |  |  |  |
|  | ***P*** | 0.006 | 0.011 |  |  | <0.01 |  |  |  |  |  |
| IL-6 | **rho** | -0.315 |  |  | 0.388 | 0.363 | 0.312 |  |  |  |  |
|  | ***P*** | 0.008 |  |  | 0.001 | 0.002 | 0.008 |  |  |  |  |
| IL-10 | **rho** |  |  |  |  |  |  |  |  |  | -0.385 |
|  | ***P*** |  |  |  |  |  |  |  |  |  | 0.001 |
| IL-17 | **rho** |  |  |  |  | -0.287 |  |  |  |  |  |
|  | ***P*** |  |  |  |  | 0.027 |  |  |  |  |  |
| Calprotectin | **rho** |  |  |  |  |  |  |  |  |  | -0.285 |
|  | ***P*** |  |  |  |  |  |  |  |  |  | 0.019 |
| Defensin | **rho** |  |  |  |  |  |  | -0.347 |  |  |  |
|  | ***P*** |  |  |  |  |  |  | 0.030 |  |  |  |
| Lysozyme | **rho** |  |  |  |  | 0.324 |  |  |  |  |  |
|  | ***P*** |  |  |  |  | 0.005 |  |  |  |  |  |
| Lactoferrin | **rho** |  |  |  |  |  | 0.313 |  |  |  |  |
|  | ***P*** |  |  |  |  |  | 0.009 |  |  |  |  |
| oxLDL | **rho** |  |  |  |  |  |  |  |  | -0.305 |  |
|  | ***P*** |  |  |  |  |  |  |  |  | 0.031 |  |
| MPO | **rho** |  |  |  |  |  |  | 0.262 |  |  |  |
|  | ***P*** |  |  |  |  |  |  | 0.028 |  |  |  |
| Vitamin D3 | **rho** |  |  |  |  |  |  |  |  |  | 0.331 |
|  | ***P*** |  |  |  |  |  |  |  |  |  | 0.040 |

IBDQ: Inflammatory Bowel Disease Questionnaire, HBI: Harvey Bradshaw index HBI, CRP: c-reactive protein, IL-: interleukin, oxLDL: oxidized low-density lipoprotein, MPO: myeloperoxidase. Spearman’s correlation test was used for the correlation analysis.
