# Supplementary material for: Clinical and inflammatory biomarkers of inflammatory bowel diseases are linked to plasma trace elements and toxic metals; new insights into an old concept
Source: Front Nutr. 2022 Dec 8;9:997356. doi: 10.3389/fnut.2022.997356 (PMC9780073; doi:10.3389/fnut.2022.997356)
Supplement: Supplementary file 5 [file Table_5.docx]

**Supplementary Table S5.** Significant correlations among trace metals and parameters reflecting disease activity in UC patients.

| **UC** | | **Manganese** | **Iron** | **Copper** | **Selenium** | **Rubidium** | **Thallium** |
| --- | --- | --- | --- | --- | --- | --- | --- |
| PMS | **rho** |  |  |  |  |  | 0.429 |
|  | ***P*** |  |  |  |  |  | 0.006 |
| CRP | **rho** |  |  | 0.447 |  |  |  |
|  | ***P*** |  |  | 0.005 |  |  |  |
| IL-6 | **rho** | -0.348 |  |  |  |  |  |
|  | ***P*** | 0.032 |  |  |  |  |  |
| IL-17 | **rho** | -0.430 |  |  |  |  |  |
|  | ***P*** | 0.010 |  |  |  |  |  |
| Lactoferrin | **rho** |  |  |  |  |  | -0.352 |
|  | ***P*** |  |  |  |  |  | 0.041 |
| oxLDL | **rho** |  |  | 0.477 |  |  |  |
|  | ***P*** |  |  | 0.014 |  |  |  |
| MPO | **rho** |  | -0.323 |  |  |  |  |
|  | ***P*** |  | 0.048 |  |  |  |  |
| Vitamin D3 | **rho** |  |  |  | 0.418 | 0.427 |  |
|  | ***P*** |  |  |  | 0.033 | 0.030 |  |

PMS: Partial Mayo score, CRP: c-reactive protein, IL-: interleukin, oxLDL: oxidized low-density lipoprotein, MPO: myeloperoxidase. Spearman’s correlation test was used for the correlation analysis.
